# Risk Factors and Outcome of HHV-6 Infections After Allogeneic Hematopoietic Cell Transplantation

**DOI:** 10.1093/ofid/ofaf383

**Published:** 2025-06-26

**Authors:** Stefanie De Vlieger, Juliette Van Hoorde, Ineke van Gremberghe, Sylvia Snauwaert, Jan Van Droogenbroeck, Tom Lodewyck, Alexander Schauwvlieghe, Dominik Selleslag, Marijke Reynders, Jens T Van Praet

**Affiliations:** Faculty of Medicine and Health Sciences, Ghent University, Ghent, Belgium; Faculty of Medicine and Health Sciences, Ghent University, Ghent, Belgium; Biostatistics Unit, Faculty of Medicine and Health Sciences, Ghent University, Ghent, Belgium; Department of Hematology, AZ Sint-Jan Brugge AV, Brugge, Belgium; Department of Hematology, AZ Sint-Jan Brugge AV, Brugge, Belgium; Department of Hematology, AZ Sint-Jan Brugge AV, Brugge, Belgium; Department of Hematology, AZ Sint-Jan Brugge AV, Brugge, Belgium; Department of Hematology, AZ Sint-Jan Brugge AV, Brugge, Belgium; Department of Medical Microbiology, AZ Sint-Jan Brugge AV, Brugge, Belgium; Faculty of Medicine and Health Sciences, Ghent University, Ghent, Belgium; Department of Nephrology and Infectious Diseases, AZ Sint-Jan Brugge AV, Brugge, Belgium

**Keywords:** allogeneic hematopoietic cell transplantation, encephalitis, HHV-6 infection, polymerase chain reaction assay

## Abstract

**Background:**

Human herpesvirus 6 (HHV-6) reactivation is frequently seen in recipients of allogeneic hematopoietic cell (HCT) transplantation, but data on clinical outcomes and risk factors are scarce. We aimed to assess the epidemiological features and outcome of clinically relevant HHV-6 infections in the first 180 days after transplantation.

**Methods:**

This was a single-center retrospective study of 405 consecutive allogeneic HCT recipients. We matched cases with a clinically relevant HHV-6 infection with control patients.

**Results:**

We found an cumulative incidence of 3.7% (95% confidence interval [CI], 1.9-5.5) and an overall incidence rate of 8.84 per 100 person-years (95% CI, 4.95-14.6) of clinically relevant HHV-6 infections during the study period. Adjusting for HCT-specific comorbidity index category, conditioning regimen, donor type, and acute graft-versus-host disease severity, the occurrence of HHV-6 encephalitis was significantly associated with a higher hazard of nonrelapse mortality (hazard ratio, 3.821; 95% CI, 1.437-10.155; *P* = .007). We observed a significantly higher mortality risk for cases compared to controls (*P* = .04) and found female sex (*P* = .017) and use of steroids (*P* = .023) or sirolimus (*P* = .006) as risk factors for HHV-6 infection. All cases had lymphopenia (<500/µL) at the day of diagnosis and 80% developed acute graft-versus-host disease around the HHV-6 infection.

**Conclusions:**

HHV-6 encephalitis remains the most detrimental disease manifestation and posttransplant factors related to immune suppression are to be included in future epidemiological studies.

## Teaser

The estimated cumulative incidence of clinically relevant HHV-6 infection at day 180 after allogeneic hematopoietic cell transplantation was 3.7%. HHV-6 encephalitis was associated with a higher hazard of nonrelapse mortality.

Recipients of an allogeneic hematopoietic stem cell transplantation (HCT) are vulnerable to a myriad of infectious complications, including the reactivation of latent herpes viridae. The human herpesvirus 6 (HHV-6), causative agent of exanthema subitum during childhood, is frequently detected in the plasma within 6 weeks after transplant and has been firmly associated with encephalitis [1]. Less well-established clinical associations of HHV-6 reactivation are nonencephalitic central nervous system dysfunction, myelosuppression, pneumonitis, hepatitis, and increased nonrelapse mortality (NRM). Risk factors for HHV-6-associated encephalitis include umbilical cord transplants, T-cell-depleted allografts, transplants from unrelated or mismatched donors, acute graft-versus-host-disease (GvHD), and treatment with glucocorticoids [2].

Observational studies on clinical outcomes of HHV-6 reactivation are scarce and have been hampered by a lack of a consensus definition of HHV-6 infection and the rarity of clinical manifestations, often focusing on encephalitis [3]. In this single-center study, we aimed to assess the epidemiological features and outcome of clinically relevant HHV-6 infections, defined as HHV-6 reactivation with moderately or strongly associated HHV-6 end-organ disease or prolonged HHV-6 viremia with weakly associated HHV-6 end-organ disease, in the first 180 days after allogeneic HCT. Furthermore, we aimed to determine risk factors and mortality hazard after HHV-6 infection.

## METHODS

### Study Population

We included 405 consecutive patients aged 18 years and older undergoing allogeneic HCT from January 2013 to June 2024 in the AZ Sint-Jan Brugge hospital. In case a patient was transplanted multiple times, we included the data from the last transplantation only. Recorded pretransplant variables were demographics (age, sex, and ethnicity), hematopoietic cell transplantation–specific comorbidity index (HCT-CI), cytomegalovirus (CMV) serostatus, human leukocyte antigen match, conditioning (categorized as myeloablative, reduced intensity [RIC] or nonmyeloablative) and GvHD prophylaxis regimens. HCT-CI scores were categorized as follows: low risk, score of 0; intermediate risk, 1-2; and high risk, 3 or more [4]. Sirolimus was only used for GvHD prevention in case there was deterioration of renal function that prohibited the use of calcineurin inhibitors. Until February 2020, monitoring for CMV was performed, after which letermovir was introduced as prophylaxis for CMV. HHV-6 polymerase chain reaction (PCR) tests were ordered on clinical indication during the study period and no systematic screening was performed. Details on the number of patients tested and the reason for testing, as well as the number and timing of follow-up PCR tests, are available as Supplementary data. The results of all HHV-6 PCR tests were assessed from admission until 180 days after HCT. This study was approved by the Ethical Committee of AZ Sint-Jan Brugge (advice number 3353).

### Definitions and Control Selection

Classification of underlying disease risk groups at transplantation, defining of the preengraftment, early and late postengraftment phase, and staging of acute GvHD was performed as described previously [5]. HHV-6 reactivation was defined as the presence of at least 1 detectable PCR in the plasma or cerebrospinal fluid. HHV-6 reactivations were considered to be clinically relevant in case there was moderately (myelosuppression or nonencephalitic central nervous system dysfunction) or strongly (encephalitis) associated HHV-6 end-organ disease, according to the 2017 European Conference On Infections in Leukaemia guidelines, or prolonged (≥2 plasma samples) HHV-6 viremia with weakly associated HHV-6 end-organ disease (pneumonitis or hepatitis) [6]. Encephalitis was defined as HHV-6 DNA in cerebrospinal fluid coinciding with acute onset of altered mental status or short-term memory loss or seizures. Pneumonitis was defined as abnormal radiographic findings with lower respiratory tract symptoms and detectable HHV-6 DNA in bronchoalveolar lavage, in the absence of another causative organism. Each case of HHV-6 infection was compared with 4 controls without evidence of HHV-6 infection, who were matched for the date of transplantation and survived at least as long as the case had before the diagnosis of HHV-6 infection. The date of diagnosis was defined as the day on which the first clinical sample positive for HHV-6 was collected. For control patients, a corresponding day was chosen based on their matched case's date of diagnosis to obtain a similar time from transplantation [7 ]. We collected detailed therapeutic and laboratory data around the date of diagnosis (details of which are provided in Supplementary Table 1).

### Microbiological Assay

Samples were tested in real time by a laboratory-developed HHV-6 PCR with the DNA polymerase gene as target, which had a lower limit of detection of 250 copies per milliliter for HHV-6B [8].

### Statistical Analyses

We aimed to study the epidemiology of HHV-6 infections by calculating incidence rates and the effect on NRM by Cox proportional hazards models. Incidence rates of HHV-6 infection episodes per 100 person-years (PY) with 95% confidence intervals (CIs) were estimated based on the exact method using the Poisson distribution (“fmsb” package). Kaplan-Meier curves were constructed using the ggsurvplot function and cumulative incidence curves using the ggcompetingrisks function (“survminer” package). Cause-specific Cox proportional hazards models were fitted for NRM, defined as death in the absence of disease relapse [9]. HHV-6 infection or encephalitis and acute GvHD were included as time-dependent covariates. Two adjusted models were fitted, each with a different approach, to include HHV-6 infection or encephalitis as a time-dependent covariate. These models further included HCT-CI category, RIC, donor type, and acute GvHD [10]. For matched case-control data, a log-rank test was used to compare survival curves between infected and noninfected patients. Independent risk factors for HHV-6 infection were identified by conditional logistic regression to account for the matching strategy, using the clogit-function. Statistical analyses were performed in SPSS version 29 and R Studio version 4.4.2.

## RESULTS

### Demographics and Baseline Characteristics

The demographics and baseline characteristics of the 405 included patients are summarized in Supplementary Table 2. The median follow-up of survivors was 68 months (range, 5-142). Ninety-six percent of the patients were Caucasian and 42% were female. Forty-seven percent of the patients were transplanted for acute leukemia. Based on HCT-CI categorization, 20% were considered low risk, 32% intermediate risk, and 43% high risk. Conditioning was mostly RIC, with myeloablative conditioning only being used in 17% of the patients. Acute GvHD grade 1 or 2 was observed in 151 patients (37%) and grade 3 or 4 in 117 patients (29%). Organs affected in patients with acute GvHD were the skin (47%), the upper gastrointestinal tract (30%), the lower gastrointestinal tract (44%), and the liver (16%).

### HHV-6 Infection Incidence and Clinical Features

**Figure 1.**
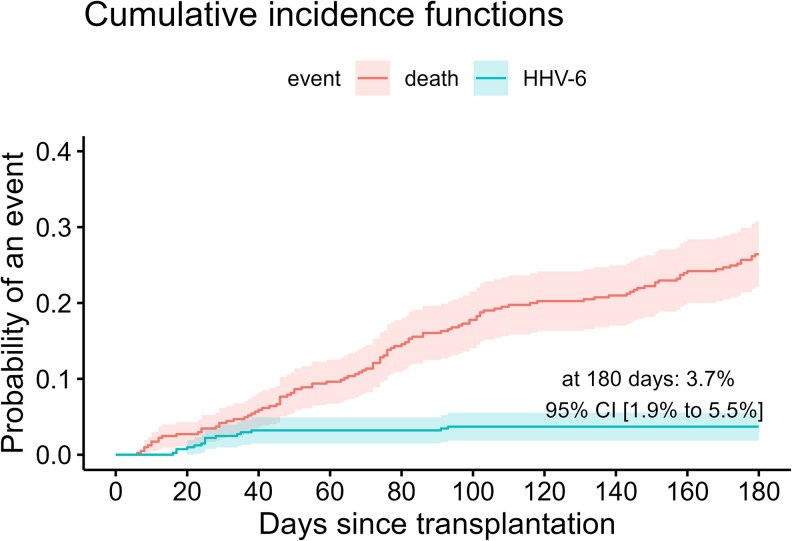
Cumulative incidence and mortality of human herpesvirus-6 (HHV-6) infection. The cumulative incidence curves of HHV-6 infection and all-cause mortality are shown. Probability with 95% confidence interval at day 180 is indicated on the graph.

During the 180 days after transplantation, 5.7% of the patients (23 of 405) experienced an episode of HHV-6 reactivation (Supplementary Figure 1). Fifteen episodes fulfilled the definition of clinically relevant HHV-6 infection. The estimated cumulative incidence of HHV-6 infection at day 180 with death as competing risk was 3.7% (95% CI, 1.9-5.5) (Figure 1). Eight patients with an HHV-6 infection had encephalitis, 4 hepatitis, 3 pneumonitis, 3 myelosuppression, and 1 nonencephalitic central nervous system dysfunction. Eleven patients were treated with ganciclovir, 2 with foscarnet, and 1 with ganciclovir and foscarnet. The overall incidence rate of HHV-6 infection during the study period was 8.84 per 100 PY (95% CI, 4.95-14.6). This rate was the same in the preengraftment phase (14.7 per 100 PY; 95% CI, 3.03-42.7) compared to the early postengraftment phase (14.8 per 100 PY; 95% CI, 7.66-25.9), with no infections occurring after day 100.

### Effect of HHV-6 Infection on MRM

The estimated cumulative incidence of NRM was 4% at day 30, 17% at day 100, and 23% at day 180. The occurrence of HHV-6 infection (hazard ratio [HR] 2.87; 95% CI, 1.323-6.226; *P* = .008) or HHV-6 encephalitis (HR 5.566; 95% CI, 2.251-13.766; *P* < .001) were significantly associated with a higher hazard of NRM in unadjusted analyses. The occurrence of HHV-6 encephalitis (HR 3.821; 95% CI, 1.437-10.155; *P* = .007) remained significantly associated after adjustment for HCT-CI category, conditioning regimen, donor type, and acute GvHD severity (Supplementary Table 3). Additional details on relapse and relapse mortality are available as Supplementary Data.

### Overall Survival After HHV-6 Infection

**Figure 2.**
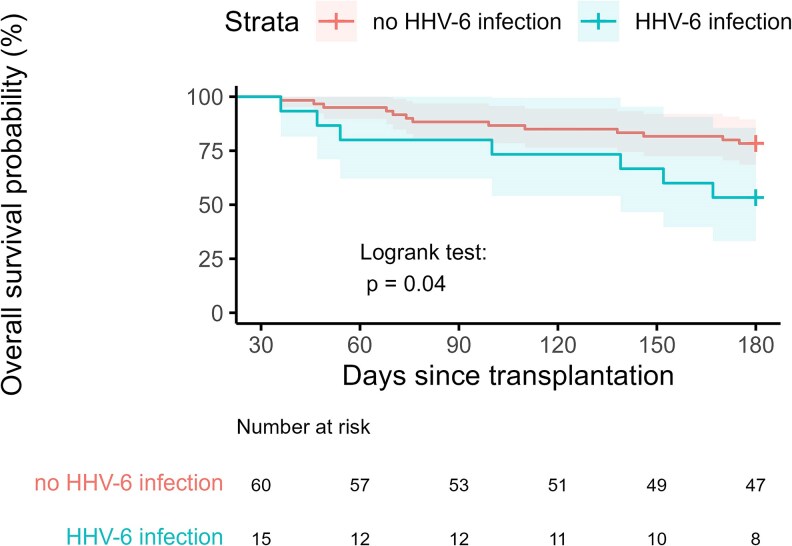
Six-month survival curves after diagnosis of human herpesvirus-6 (HHV-6) infection occurring after allogeneic stem cell transplantation (SCT). Survival of patients with HHV-6 infection and matched control patients was compared by a log-rank test.

Seven patients died within the first year after HHV-6 infection, resulting in an estimated 1-year mortality of 47% (95% CI, 20-73). Median time to death of these patients was 46 days (range, 12-147). The causes of death are available as Supplementary Data. Figure 2 shows the probability of overall survival of patients with HHV-6 infection compared to matched controls. We observed a significantly higher mortality risk for cases compared to controls (*P* = .04).

### Risk Factors for HHV-6 Infection

Unadjusted analyses comparing cases and controls indicated a significantly higher risk for female patients (*P* = .017) and patients using steroids (*P* = .023) or sirolimus (*P* = .006) (Supplementary Tables 1 and 4). Furthermore, all patients with HHV-6 had lymphopenia (<500/µL) at the day of diagnosis and 80% developed acute GvHD around the HHV-6 infection. In 42% of the patients with cooccurrence of both conditions, HHV-6 preceded the development of acute GVHD. Additional details on all studied risk factors and the timing of HHV-6 occurrence compared to the onset of acute GvHD are available as Supplementary Data. Supplementary Figure 2 shows the difference between the day of onset of HHV-6 infection and the occurrence of GvHD for patients who developed both conditions.

## DISCUSSION

Although reactivation of HHV-6 after allogeneic HCT is frequently observed, its clinical associations and outcome of patients with disease symptoms are less well understood. Including reactivations with moderately or strongly associated HHV-6 end-organ disease and prolonged HHV-6 viremias with weakly associated HHV-6 end-organ disease, we found that 3.7% of an adult patient series developed HHV-6 infection in the first 180 days after transplantation. We observed an equal incidence in the preengraftment and early postengraftment phase, finding no infections afterward. These observations confirm that future studies on the prevention and treatment of clinically relevant HHV-6 infections should focus on the earliest phase after transplant.

Adverse outcomes have been reported in HCT recipients with HHV-6 encephalitis. We found a significant association of HHV-6 encephalitis, but not HHV-6 infection, with NRM, independent of classical risk factors associated with NRM. Matching cases to controls without evidence of HHV-6 infection, we found a significantly higher overall mortality in patients after HHV-6 infection. Their causes of death were not directly linked to HHV-6 but rather suggested an indirect effect of HHV-6 on mortality, potentially by immune dysregulation. Our observations are in line with a recent meta-analysis [11] that reported higher NRM and overall mortality after HHV-6 reactivation and suggest that HHV-6 encephalitis remains the most detrimental disease manifestation.

Using a case-control design, we found a significantly higher risk for HHV-6 infection in patients with female sex and ongoing use of steroid or sirolimus. Furthermore, all patients with HHV-6 infection had lymphopenia (<500/µL) and 80% developed GvHD around the HHV-6 diagnosis. Previous studies have reported HCT recipients receiving transplants from mismatched or unrelated donors, and those receiving myeloablative conditioning to be at higher risk [12, 13]. We also observed that these groups were at higher risk, without reaching statistical significance, potentially because of a type II statistical error. In line with our findings, several studies have identified ongoing receipt of glucocorticoids as risk factor [13, 14]. Furthermore, several studies have suggested an association between HHV-6 and acute GvHD [15]. Our data showed that these conditions often cooccurred, but the difference of timing of onset of both conditions did not hint toward a causative effect of one to another. The association between HHV-6 infection and sirolimus use has not been described previously. Because sirolimus was used in patients with deterioration of renal function, prohibiting the use of calcineurin inhibitors, this observation warrants confirmation in future studies. Potentially, the renal impairment itself might act as a confounder as we did not include data on acute kidney injury in the analysis. In contrast to our findings, other series have found higher a incidence of HHV-6 reactivation in men [12], which might be related to differences in the definition of HHV-6 infection. Collectively, our findings support that posttransplant factors related to immune suppression are to be included in future studies looking into risk factors of this early posttransplant opportunistic infection.

Limitations of this study include its retrospective design and inclusion of patients in only 1 single center. Since screening for HHV-6 reactivation was not performed systematically but on clinical indication, episodes of reactivation might have been missed. Also, we did not formally rule out HHV-6 reactivation in control patients as systematic screening by PCR was not performed. Finally, we were unable to fit a valid multivariable conditional logistic regression model of potential risk factors.

## Supplementary Material

ofaf383_Supplementary_Data

## Supplementary Data

Supplementary materials are available at *Open Forum Infectious Diseases* online. Consisting of data provided by the authors to benefit the reader, the posted materials are not copyedited and are the sole responsibility of the authors, so questions or comments should be addressed to the corresponding author.
