# Supplementary material for: Risk Factors and Outcome of HHV-6 Infections After Allogeneic Hematopoietic Cell Transplantation
Source: Open Forum Infect Dis. 2025 Jun 26;12(7):ofaf383. doi: 10.1093/ofid/ofaf383 (PMC12272341; doi:10.1093/ofid/ofaf383)
Supplement: ofaf383_Supplementary_Data [file ofaf383_supplementary_data.zip › De Vlieger_2025_ Supplementary_data.docx]

**Risk factors and outcome of HHV-6 infections after allogeneic hematopoietic cell transplantation**

Stefanie De Vlieger, Juliette Van Hoorde, Ineke van Gremberghe, Sylvia Snauwaert, Jan Van Droogenbroeck, Tom Lodewyck, Alexander Schauwvlieghe, Dominik Selleslag, Marijke Reynders, Jens T. Van Praet

**Number and indication of PCR tests performed in patients without HHV-6 infection**

Of the 405 included patients, 47 (12%) had at least one PCR test for HHV-6 (plasma, bronchoalveolar fluid or cerebrospinal fluid). For the patients (n = 32) with a negative test or not fulfilling the definition of HHV-6 infection, reasons for testing were encephalopathy/encephalitis (n=10), graft failure (n=7), rash/fever (n=6), pneumonitis (n=5) and hepatitis (n=4).

**Number and timing of follow-up PCR tests in patients with HHV-6 reactivation**

The mean number of follow-up PCR tests was 5 (range 1-23). The mean frequency of testing was 1.69 tests/week (range 0.39 – 7.0).

**Effect of HHV-6 infection on non-relapse mortality**

Of the 405 included patients, 114 died within the first 180 after transplantation: 19 after relapse and 95 without. There were 37 relapses in the first 180 days. The estimated cumulative incidence of relapse mortality was 0% at day 30, 2% at day 100, and 5% at day 180.

**Overall survival after HHV-6 infection**

The causes of death of the 7 patients who died within 1 year after diagnosis of HHV-6 were refractory graft-versus-host-disease (n=3), septic shock due to intestinal ischemia (n=1), respiratory failure and encephalopathy (n=1), septic shock and respiratory failure (n=1) and refractory EBV reactivation (n=1). None of these patients underwent autopsy.

**Supplementary Tables**

Supplementary Table 1 Clinical and biological characteristics of 15 allogeneic HCT recipients with HHV-6 infections (cases) and 60 control HCT recipients without evidence of HHV-6 infection

|  | **Cases (n = 15)** | **Controls (n = 60)** |
| --- | --- | --- |
| **Age – no. (%)**  **<50**  **50-65**  **>65** | 2 (13)  3 (20)  10 (66) | 14 (23)  18 (30)  28 (47) |
| **Sex, female – no. (%)** | 11 (73) | 21 (35) |
| **HCT-CI – no. (%)**  **0**  **1-2**  **>3** | 6 (40)  6 (40)  3 (20) | 14 (23)  13 (22)  30 (50) |
| **Multiple transplants – no. (%)** | 2 (13) | 1 (1.7) |
| **Diagnosis – no. (%)**  **Acute lymphoid leukemia**  **Acute myeloid leukemia**  **Lymphoma**  **MDS-MPN-CML-other** | 3 (20)  6 (40)  1 (7)  5 (33) | 7 (12)  22 (37)  3 (5)  28 (47) |
| **Donor type – no. (%)**  **Sibling**  **MUD**  **Haplo** | 1 (7)  10 (67)  4 (27) | 16 (27)  37 (62)  7 (12) |
| **Conditioning – no. (%)**  **Standard**  **RIC**  **Non-myeloablative** | 1 (7)  13 (87)  1 (7) | 11 (18)  46 (77)  3 (5) |
| **T cell–depleted transplant – no. (%)** | 10 (67) | 36 (60) |
| **Active aGvHD* – no. (%)** | 7 (46) | 14 (23) |
| **Neutropenia (< 500/μl)* – no. (%)** | 3 (20) | 11 (18) |
| **Lymphopenia (< 500/μl)* – no. (%)** | 15 (100) | 49 (82) |
| **Monocytopenia (< 500/μl)* – no. (%)** | 10 (67) | 26 (43) |
| **Steroid use* – no. (%)** | 9 (60) | 17 (28) |
| **Sirolimus use (versus cyclosporin)* – no. (%)** | 13 (87) | 24 (40) |
| **Mycophenolate mofetil use* – no. (%)** | 6 (40) | 27 (45) |
| **Ruxolitinib use* – no. (%)** | 1 (7) | 2 (3) |
| **CMV reactivation – no. (%)** | 0 (0) | 6 (10) |

HCT-CI denotes hematopoietic stem cell transplant–specific comorbidity index, RIC reduced intensity conditioning, HL Hodgkin lymphoma, MDS Myelodysplastic syndrome, MPN Myeloproliferative neoplasmata, CML chronic myeloid leukemia, aGvHD acute graft versus host disease. All variables are reported at the time of HHV6 infection for cases or matched time for controls, unless otherwise stated. *At the time of transplant

Supplementary Table 2 baseline characteristics and demographic of allogeneic HCT recipients (n=405)

| Age – mean (SD)  Male  Race – no. (%)  Caucasian  Non-Caucasian Disease – no. (%)  Acute leukemia  Hodgkin lymphoma  Myelodysplastic syndrome  Myeloproliferative disorder  Non-Hodgkin lymphoma  Other Disease risk – no. (%)  High  Standard HCI-CI score – median (IQR) HCI-CI score – no. (%)  0  1-2  ≥3  missing  GvHD prophylaxis – no. (%)  CNI  CNI and MMF  CNI, MMF and cyclophosphamide  CNI and MTX  Other T cell–depleted transplant – no. (%)  No  Yes Conditioning regimen – no. (%)  Myeloablative  Reduced intensity conditioning  Non-myeloablative HLA match – no. (%)  Haploidentical related  Matched unrelated  Matched related Donor CMV status – no. (%)  Negative  Positive Recipient CMV status– no. (%)  Negative  Positive | 55.9 (13.7)  236 (58.3)  390 (96.2)  15 (3.7)  189 (46.6)  8 (1.97)  107 (26.4)  51 (12.6)  22 (5.43)  28 (6.91)  267 (65.9)  138 (34.1)  2 (2.0)  79 (19.5)  128 (31.6)  173 (42.7)  25 (6.2)  167 (41.2)  144 (35.6)  26 (6.4)  34 (8.4)  34 (8.4)  183 (45.2)  222 (54.8)  70 (17.3)  322 (79.5)  13 (3.2)  36 (8.9)  235 (58)  134 (33.1)  279 (68.9)  126 (31.1)  242 (59.8)  163 (40.2) |
| --- | --- |

HCT denotes hematopoietic stem cell transplant, IQR interquartile range, SD standard deviation, HCT-CI hematopoietic stem cell transplant–specific comorbidity index, GvHD graft versus host disease; CNI calcineurin inhibitor, MMF mycophenolate mofetil, MTX methotrexate, CMV cytomegalovirus.

Supplementary Table 3 Results from two multiple cause-specific Cox proportional hazards models for non-relapse mortality

| **Model** | **Variables** | **HR (95% CI)** | **P value** |
| --- | --- | --- | --- |
| **HHV6 infection** | HHV6 infection | 1.515 (0.661-3.471) | 0.326 |
|  | HCT-CI 0 | Reference |  |
|  | HCT-CI 1-2 | 1.007 (0.519-1.925) | 0.984 |
|  | HCT-CI 3 of higher | 1.617 (0.893-2.930) | 0.113 |
|  | Myeloablative conditioning | Reference |  |
|  | RIC | 2.089 (0.996-4.379) | 0.051 |
|  | Non-myeloablative conditioning | 2.609 (0.766-8.886) | 0.125 |
|  | Matched related | Reference |  |
|  | Matched unrelated | 1.660 (0.965-2.855) | 0.067 |
|  | Haploidentical related | 2.192 (1.026-4.685) | 0.043 |
|  | Absent aGvHD | Reference |  |
|  | aGvHD grade I-II | 0.656 (0.288-1.493) | 0.315 |
|  | aGvHD grade III-IV | 7.159 (4.072-12.585) | <0.001 |
| **HHV6 encephalitis** | HHV6 encephalitis | 3.821 (1.437-10.155) | 0.007 |
|  | HCT-CI 0 | Reference |  |
|  | HCT-CI 1-2 | 1.135 (0.576-2.235) | 0.714 |
|  | HCT-CI 3 of higher | 1.746 (0.963-3.165) | 0.066 |
|  | Myeloablative conditioning | Reference |  |
|  | RIC | 2.007 (0.957-4.211) | 0.065 |
|  | Non-myeloablative conditioning | 2.615 (0.768-8.903) | 0.124 |
|  | Matched related | Reference |  |
|  | Matched unrelated | 1.602 (0.932-2.756) | 0.088 |
|  | Haploidentical related | 2.399 (1.141-5.043) | 0.021 |
|  | Absent aGvHD | Reference |  |
|  | aGvHD grade I-II | 0.646 (0.283-1.472) | 0.298 |
|  | aGvHD grade III-IV | 7.073 (4.022-12.441) | <0.001 |

CI denotes confidence interval, HCT-CI hematopoietic stem cell transplant–specific comorbidity index, RIC reduced intensity conditioning, aGvHD acute graft versus host disease.

Supplementary Table 4 Results from simple conditional logistic regression

| **Characteristic*** | **Categories** | **HR (95% CI)** | **P value** |
| --- | --- | --- | --- |
| **Age** | <50 | Reference |  |
|  | 50-65 | 2.55 (0.46-14.1) | 0.3 |
|  | >65 | 1.29 (0.18-9.35) | 0.8 |
| **Sex** | Male | Reference |  |
|  | Female | 4.34 (1.30-14.5) | 0.017 |
| **HCT-CI** | 0 | Reference |  |
|  | 1-2 | 1.15 (0.3-4.41) | 0.8 |
|  | 3 of higher | 0.23 (0.05-1.01) | 0.052 |
| **Multiple transplants** | No | Reference |  |
|  | Yes | 8.0 (0.73-88.2) | 0.09 |
| **Diagnosis** | ALL | Reference |  |
|  | AML | 0.65 (0.12-3.5) | 0.6 |
|  | Lymphoma | 0.8 (0.06-11.3) | 0.9 |
|  | MDS-MPN-CML-other | 0.44 (0.09-2.17) | 0.3 |
| **Donor type** | Matched related | Reference |  |
|  | Matched unrelated | 4.02 (0.5-32.4) | 0.2 |
|  | Haploidentical related | 10.6 (0.9-125) | 0.06 |
| **Conditioning** | Standard | Reference |  |
|  | RIC | 3.01 (0.37-24.7) | 0.3 |
|  | Non-myeloablative | 3.83 (0.18-81.2) | 0.4 |
| **T cell–depleted transplant** | No | Reference |  |
|  | Yes | 1.29 (0.42-3.99) | 0.7 |
| **Active aGvHD**** | No | Reference |  |
|  | Yes | 2.93 (0.88-9.78) | 0.081 |
| **Neutropenia (< 500/μl)**** | No | Reference |  |
|  | Yes | 1.11 (0.27-4.61) | 0.9 |
| **Monocytopenia (< 500/μl)**** | No | Reference |  |
|  | Yes | 2.31 (0.76-7.04) | 0.14 |
| **Steroid use**** | No | Reference |  |
|  | Yes | 6.59 (1.3-33.4) | 0.023 |
| **Cyclosporin or sirolimus use**** | Cyclosporin | Reference |  |
|  | Sirolimus | 8.97 (1.90-42.4) | 0.006 |
| **Mycophenolate mofetil use**** | No | Reference |  |
|  | Yes | 0.79 (0.23-2.70) | 0.7 |
| **Ruxolitinib use**** | No | Reference |  |
|  | Yes | 2.45 (0.14-42.6) | 0.5 |

CI denotes confidence interval, HCT-CI hematopoietic stem cell transplant–specific comorbidity index, RIC reduced intensity conditioning, aGvHD acute graft versus host disease. All variables are reported at the time of HHV6 infection for cases or matched time for controls, unless otherwise stated. *For lymphopenia and CMV reactivation, a reliable conditional logistic model could not be fitted given all cases had lymphopenia and none had CMV reactivation. **At the time of transplant

**Supplementary Figure**

Supplementary Figure 1

Flowchart of patient inclusion

Supplementary Figure 2

The frequency of the difference in days between the day of the onset of HHV-6 infection and the occurrence of graft-versus-host-disease is shown for the 12 patients who developed both conditions.
