# Supplementary material for: Risk Factors and Outcome of HHV-6 Infections After Allogeneic Hematopoietic Cell Transplantation
Source: Open Forum Infect Dis. 2025 Jun 26;12(7):ofaf383. doi: 10.1093/ofid/ofaf383 (PMC12272341; doi:10.1093/ofid/ofaf383)
Supplement: ofaf383_Supplementary_Data [file ofaf383_supplementary_data.zip › De Vlieger_2025_Supplementary_Figure_1.pptx]

## Slide 1
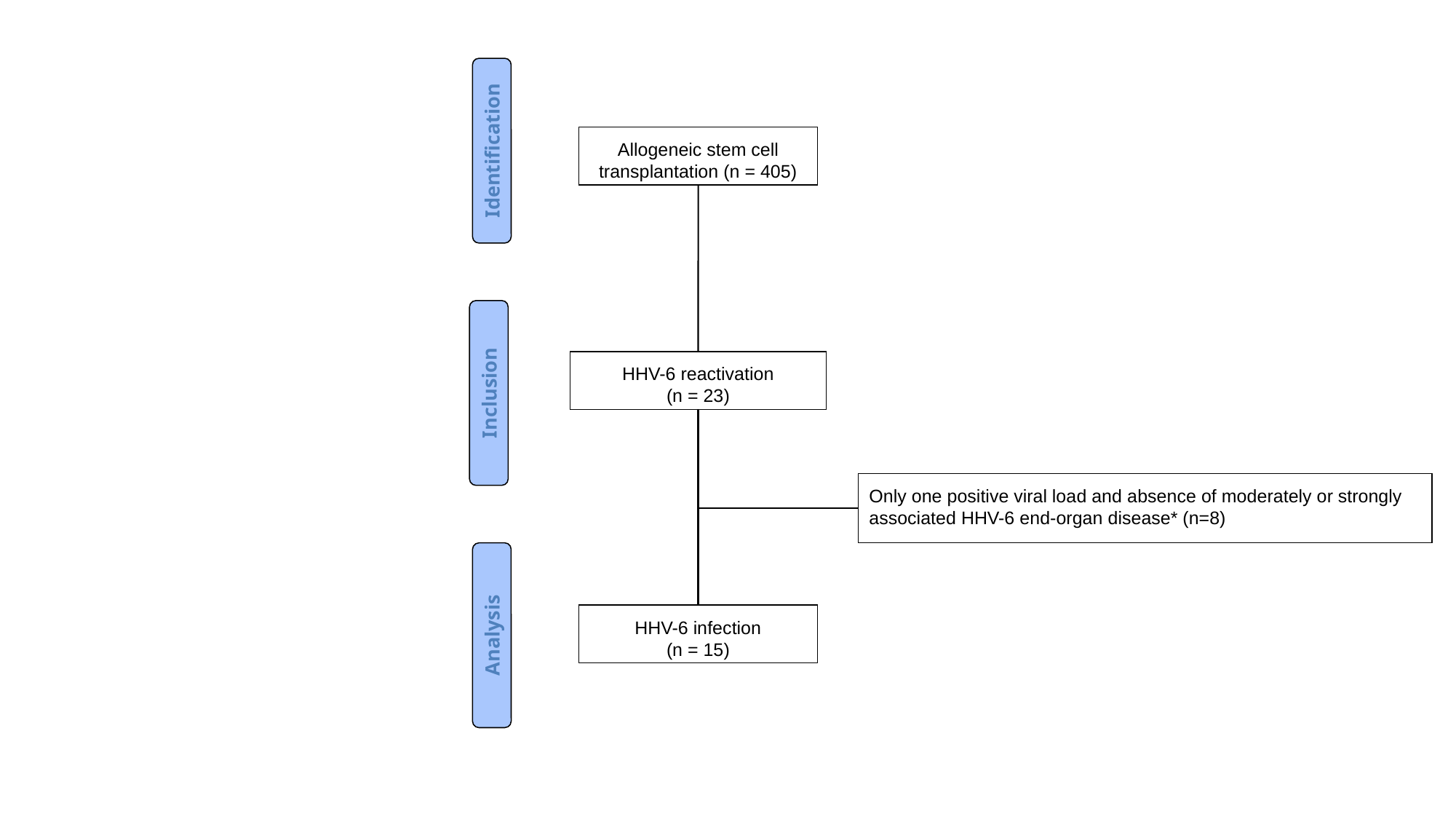

Allogeneic stem cell transplantation (n = 405)
Identification
HHV-6 reactivation
(n = 23)
Inclusion
Only one positive viral load and absence of moderately or strongly associated HHV-6 end-organ disease* (n=8)
HHV-6 infection
(n = 15)
Analysis
