# Supplementary figures and images for: Risk Factors and Outcome of HHV-6 Infections After Allogeneic Hematopoietic Cell Transplantation
Source: Open Forum Infect Dis. 2025 Jun 26;12(7):ofaf383. doi: 10.1093/ofid/ofaf383 (PMC12272341; doi:10.1093/ofid/ofaf383)

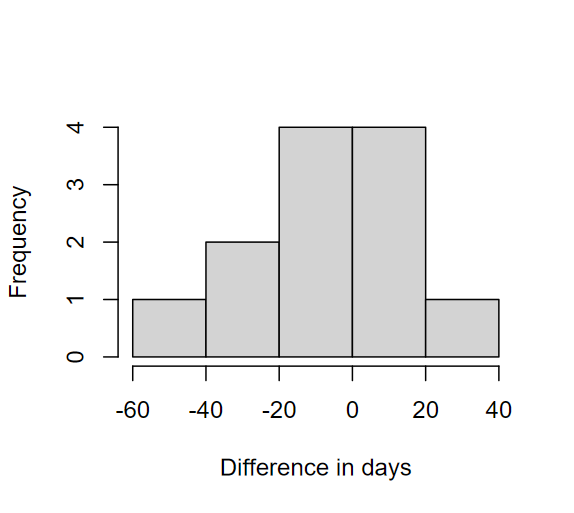

Supplement: ofaf383_Supplementary_Data [file ofaf383_supplementary_data.zip › De Vlieger_2025_Supplementary_Figure_2.tiff]
